# Impact of Adenotonsillectomy on Weight Gain in Children: A Systematic Review

**DOI:** 10.3390/children12030270

**Published:** 2025-02-23

**Authors:** Omar Ibrahim Alanazi, Abdulaziz Alsharif, Arwa Alsharif, Hanan Ismail Wasaya, Faten Aljifri, Atheer Mohammed, Reem Halawani, Abdalhadi Mahmood Halawani, Baraa Awad, Mohammed Halawani

**Affiliations:** 1 College of Medicine, King Saud University, Riyadh 11426, Saudi Arabia; alanazi323@ksau-hs.edu.sa; 2 Department of Medicine and Surgery, Vision College, Jeddah 23643, Saudi Arabia; 202313034@vision.edu.sa; 3 Department of Medicine and Surgery, Batterjee Medical College, Jeddah 21442, Saudi Arabia; 140204.hanan@bmc.edu.sa (H.I.W.); 130066.faten@bmc.edu.sa (F.A.); 140278.atheer@bmc.edu.sa (A.M.); 140112.reem@bmc.edu.sa (R.H.); 21110550@bmc.edu.sa (A.M.H.); 4 Department of Otolaryngology-Head & Neck Surgery, College of Medicine, King Saud bin Abdulaziz University for Health Sciences, Jeddah 21423, Saudi Arabia; awadba@ngha.med.sa; 5 King Abdullah International Medical Research Center, Ministry of the National Guard—Health Affairs, Jeddah 22384, Saudi Arabia; 6 Pediatric ENT, King Abdullah Specialized Children’s Hospital (KASCH), Riyadh 11426, Saudi Arabia; halawanimo1@mngha.med.sa; 7 Ministry of National Guard Health Affairs (MNGHA), Riyadh 11426, Saudi Arabia; 8 King Abdullah International Medical Research Center, Riyadh 11426, Saudi Arabia

**Keywords:** adenotonsillectomy, weight gain, obstructive sleep apnea, pediatric, growth outcomes, body mass index

## Abstract

**Background**: Adenotonsillectomy (AT) is a common surgical procedure among pediatrics, usually performed to treat obstructive sleep apnea (OSA), recurrent tonsillitis, and chronic adenoid hypertrophy. The aim of our systematic review is to evaluate the relationship between AT and postoperative weight gain in children to guide clinicians in optimizing surgical outcomes. **Methods**: A systematic search was conducted following the PRISMA guidelines in PubMed, MEDLINE, and Web of Science databases. Studies evaluating weight, BMI, and growth parameters before and after AT were included. Data were collaboratively extracted, including patient demographics, baseline weight status, comorbidities, and long-term outcomes. **Results**: Underweight children (less than the 3rd percentile on the growth chart) who underwent AT often experienced “catch-up growth” in weight, while obese children (above the 95th percentile on the growth chart) had postoperative weight gain that exacerbated pre-existing obesity. These outcomes were affected by factors such as baseline weight, age, and comorbid conditions. **Conclusions**: AT can improve the quality of life in underweight children, while overweight or obese children need careful monitoring and nutrition counseling postoperatively to mitigate excessive weight gain. More randomized trials are needed to better understand the metabolic and growth implications of AT and to refine clinical guidelines.

## 1. Introduction

Adenotonsillectomy (AT) is one of the most commonly performed surgical procedures in the pediatric population. It is predominantly indicated for treating obstructive sleep apnea (OSA), recurrent tonsillitis, and chronic adenoid hypertrophy [1]. This surgical intervention has been demonstrated to considerably ameliorate airway obstruction, sleep quality, and quality of life in affected children [2]. Nevertheless, the last few years have been characterized by studies reporting an association between AT and weight gain after surgery in the pediatric population, attracting wide attention and controversy [3].

The postoperative weight gain reported after AT has been related to improved feed efficiency with decreased obstruction, better quality sleep with increased growth hormone activity, and metabolic/feeding habit changes [4]. For underweight children (less than the 3rd percentile on the growth chart), weight gain is a goal; however, excessive gain should be a concern in children who had a healthy weight or were overweight before surgery (above the 95th percentile on the growth chart) [5]. The association between AT and weight gain has implications for not only surgical outcomes but also long-term pediatric health outcomes, including non-infectious respiratory disease, metabolic syndrome, cardiovascular disease, and psychosocial health. Despite increased awareness, the literature on the topic is equivocal, with studies presenting differing findings depending on baseline weight status, age, gender, or presence of comorbidities [6]. Notably, variances in study designs and definitions of weight gain also make it difficult to interpret this association.

This systematic review aims to analyze the evidence of the effect of AT on weight gain in the pediatric population by identifying trends, risk factors, and long-term consequences. This review aims to summarize the available data to provide data-driven evidence to assist clinicians in preoperative counseling, postoperative monitoring, and general management of the pediatric population undergoing AT.

## 2. Materials and Methods

### 2.1. Search Strategy

This systematic review was performed according to the PRISMA (Preferred Reporting Items for Systematic Reviews and Meta-Analyses) guidelines and was prospectively registered in PROSPERO (CRD42025634918) (Appendix A) [7]. An electronic search of the databases MEDLINE, PubMed, and Web of Science was performed without temporal boundaries. Authors M.H. and A.A. developed a search strategy, which has been approved by the rest of the study team. In exploring the effects of AT on childhood weight gain, relevant studies were identified with broad inclusion criteria using a combination of Medical Subject Headings (MeSHs) terms including “Adenotonsillectomy” OR “Pediatric Adenotonsillectomy” OR “Tonsillectomy” OR “Adenoidectomy” AND “Weight Gain” OR “BMI Change” OR “Postoperative Growth” AND “Pediatric” OR “Children” AND “Outcomes” OR “Complications”. A review of the references to the studies was also performed to identify any missing papers.

The search method included several databases: PubMed (*n* = 3785), MEDLINE (*n* = 2074), and Web of Science (*n* = 5574). First, the records were checked for duplicates, yielding 11,433 unique entries. During the eligibility phase, we assessed 4479 records and excluded 6954 records by using the predefined criteria. Out of the reviewed records, 1320 full-text articles were evaluated for eligibility, and 3159 were excluded for detailed reasons. Seventy papers met the inclusion criteria for qualitative synthesis.

### 2.2. Study Selection

#### 2.2.1. Inclusion Criteria

This systematic review included studies examining the impact of AT on weight gain in children ages 0 to 18 years. The studies included preoperative and postoperative weight measurements (e.g., weight, body mass index (BMI), and growth percentiles) to evaluate weight changes after AT. We included quasi-experimental studies, randomized controlled trials (RCTs), cohort studies, case-control studies, and observational studies published in the English language. This review included papers published in peer-reviewed journals or other quality sources.

#### 2.2.2. Exclusion Criteria

The current systematic review excluded studies that do not evaluate the impact of AT on weight gain in the pediatric population. It also omitted research involving anyone older than 18. Analyses of surgical management unrelated to AT, animal studies, and in vitro studies were excluded. We excluded studies in any language other than English and ones that did not report sufficient data, specifically those where AT bidding outcomes, exact weights resulting from timetable changes, or relevant statistical analysis components required for judging the impact of AT on total weight gain were missing.

#### 2.2.3. Screening and Data Extraction

The records were organized and safely archived for systemic review, and access was limited explicitly to the research team. The remaining findings were then imported to Rayyan for screening based on relevance and for title and abstract screening by four writers (O.I.A., A.A., H.I., and F.A.) (https://www.rayyan.ai/, accessed on 8 November 2024). Then four authors (A.M., R.H., B.A., and A.M.H.) performed a full-text assessment of studies that were positive in the first stage of filtering for inclusion or exclusion [8]. Previous conversations with M.H. and other researchers helped resolve any differences in the screening process. A.A., A.A., and M.H. developed an Excel spreadsheet to extract the following data from selected studies: title, author name, country, year of publication, journal name, study design, level of evidence, sample size, preoperative and postoperative weight data, surgical complication, and long-term outcomes.

#### 2.2.4. Quality Assessment and Bias Evaluation

To assess each study for risk of bias and the quality of evidence from included studies, we used the Grading of Recommendations Assessment, Development, and Evaluation (GRADE) approach [9]. This detailed review evaluated the quality of the evidence across all research and subsequently calculated an overall quality score which was interpreted as a risk of bias. Both retrospective and prospective cohort study biases were assessed using the Newcastle–Ottawa Scale (Appendix B) [10]. We assessed the risk of bias for the randomized controlled trials (RCTs) using the updated Cochrane Risk of Bias tool for randomized trials (RoB 2) (Appendix D) [11]. The non-randomized studies included in this review were assessed using the MINORS criteria (Appendix C) [12]. This type of evaluation provides insight into both the quality of the included research and potential biases in the studies, thus increasing the reliability of the findings presented.

### 2.3. Data Synthesis

Conducting a meta-analysis was not possible due to excessive heterogeneity and high degrees of inconsistency in data types. Notably, this heterogeneity was further exemplified by the range of study methodologies, e.g., RCT, cohort study, or observational study, each applying different types of outcome assessments postoperatively. The studies varied in the characteristics of the patients studied, such as age groups, baseline weight status, and comorbid disorders, like obstructive sleep apnea or recurrent tonsillitis. These heterogeneities impact surgical outcomes and complicate inter-study comparisons. It also prevented us from pooling results into a single meta-analysis.

Although diversity across studies limited the generalizability of some findings, it also highlighted the complexity of evaluating the effect of AT on weight gain. There is variability in such outcomes and thus future studies should be performed utilizing standardized techniques and outcome measures.

## 3. Results

A series of searches in each electronic database yielded 11,433 articles. Then, 6954 duplicates were removed, and inclusion and exclusion criteria were applied. Of these, 3159 papers were excluded for the following reasons: full text not available, repeated articles, insufficient methodological quality, other surgical procedures included, and not studying the problem of AT. Finally, we excluded those studies not conducted in English and studies without adequate data to analyze the effect of weight on AT. All these references were reviewed, and 1320 articles were then assessed for eligibility. Seventy publications met all criteria for this systematic review of adenotonsillectomy effects on weight gain in children and were evaluated further.

**Figure 1:**
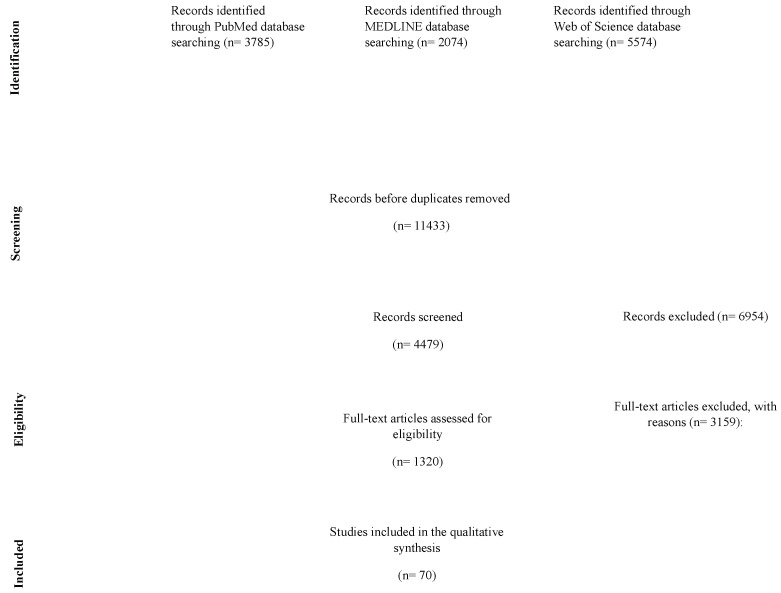
The detailed PRISMA chart used for this systematic review, delineating the many stages of this study’s selection process.

Randomized controlled trials, cohort studies, and observed studies were among the included paper types. This heterogeneity allowed us to better estimate the impact of AT on children’s weight gain. The PRISMA flow chart process diagram is shown in Figure 1. The 38 extracted studies, published between 1988 and 2024, were from diverse global regions.

The articles that reference the effect of AT on weight gain in children, as well as characteristics of their cohort, are shown in Table 1.

### 3.1. Patients’ Profiles and Characteristics

This systematic review included a total of 8424 patients undergoing AT. The ages of study participants varied considerably, ranging from 12 months to adolescents up to 18 years, reflecting the diverse conditions and patient demographics across the studies. We also observed a gender imbalance, with females having more AT surgeries than males. A pooled analysis of studies reported a male–female ratio of about 2:1 in childhood populations [31].

**Figure 2:**
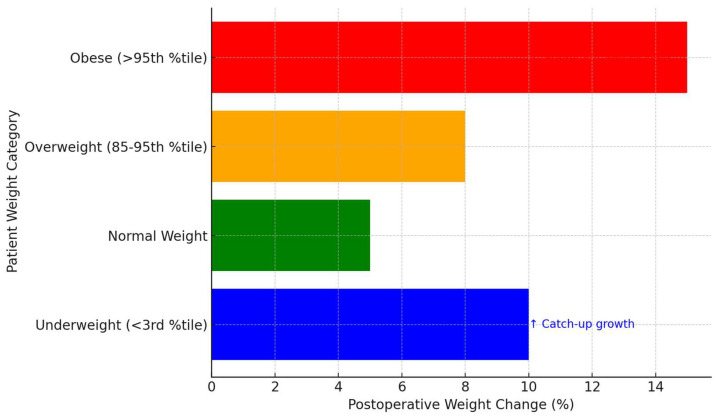
Postoperative weight changes in children undergoing adenotonsillectomy.

Weight gain following AT was a prominent outcome, particularly in underweight (less than the third percentile on the growth chart) or normal-weight children as shown in Figure 2. A retrospective cohort study revealed that thin children (aged 12–18 years) showed a significant increase in BMI z-score (from −2.4 to −0.59, *p* = 0.046). On the other hand, overweight and obese children (above the 95th percentile on the growth chart) were trending towards normalization, with small reductions in BMI z-scores (overweight: from 1.508 to 1.48; obese: from 2.288 to 2.00, *p* = 0.06) [32]. Regarding surgical techniques, a study that compared between intracapsular tonsillotomy (ICT) and extracapsular tonsillectomy (ECT) revealed distinct outcomes. They found that both techniques improved respiratory symptoms and quality of life, as indicated by reductions in OSA-18 survey scores and improvements in pulse oximetry results. However, ICT shows advantages in decreasing postoperative morbidity, including pain and bleeding, while ECT showed a more definitive removal of tonsillar tissue, mitigating the risks of regrowth [33].

Overall, these findings highlight the need for tailored perioperative management based on the child’s baseline weight status, age, and comorbid conditions. Further research into the long-term metabolic and growth outcomes after AT is warranted to refine treatment strategies and optimize patient care.

### 3.2. Impact of Adenotonsillectomy on Postoperative Growth Outcomes

This systematic review provides a comprehensive analysis of studies on neonates and infants undergoing AT, focusing on postoperative outcomes related to weight, BMI, and growth over follow-up periods ranging from 2 to 12 months. One study found that children aged 6 to 36 months with OSA improved a lot after AT. 75% of those ninety-nine patients saw improvement in weight percentiles, and 65% gained 15 kg or more (*p* < 0.001), illustrating the impact of resolving upper airway obstruction on growth [34]. Similarly, another study indicates substantial growth in children after AT, with an average height increase of 6.66 cm (*p* = 0.0004) and weight gain of 2.15 kg (*p* = 0.0010) over six months, emphasizing the benefits of addressing pharyngopalatine hypertrophy [35].

Moreover, a study that investigated the growth factors in prepubertal children after AT found a different result. They found that there are significant increases in height, weight, and BMI z-scores among normal- and under-growth children (*p* < 0.05), while obese children showed significant improvements only in height z-scores (*p* = 0.028). These findings underscore how AT promotes growth while minimizing excessive weight gain, with the severity of sleep disturbance being identified as a predictor of improved height [36]. Additional details on the impact of AT on weight gain, along with pre- and post-surgical weight/BMI metrics, are summarized in Table 2.

## 4. Discussion

We conducted a systematic review to examine how AT affects children’s growth and weight outcomes. We find that the impact was different for those who had different initial weights. Our findings demonstrate a strong association between AT and improvements in growth parameters, including weight gain, increases in BMI, and height z-scores. This review emphasizes the clinical significance of AT, especially for promoting “catch-up growth” in children of low body weight and the ethereal postoperative physiological effects of sleep, all of which contribute to growth outcomes. These findings further underline the importance of individualized surgery and comprehensive perioperative care, especially for children with comorbidities or initial weight problems, in order to promote recovery and long-term health.

The findings of this systematic review underscore the multifaceted impact of AT on pediatric patients, especially in terms of postoperative weight gain, changes in BMI, and growth outcomes [39,54]. This review also confirms that AT results in clear improvements across the board for children with OSA or chronic tonsillitis [44,45]. This is important not only in its own right but also as an indication of greater upward movement in growth standards. These various opinions on the question should speak for themselves, but with most papers showing final height z-scores higher than pre-“adenoids take” ones and the majority tending to be primary-growth patterns, lateral drift toward normative growth is an evident trend that is consistent with our findings [55,56].

Children aged 6–36 months with OSA displayed overall substantial “catch-up growth”, with 75% achieving higher weight percentiles and 65% recording big weight gains (*p* < 0.001) [34,57]. These findings stress the importance of alleviating obstructions of the upper airway to support children’s optimal growth trajectories [58,59]. However, the review also points to the complexity of weight outcomes for children with different initial weights. Although underweight and normal-weight children benefit from improved growth, obese children see only limited improvements, with significant increases occurring in height z-scores alone (*p* = 0.028) [36,60]. This split means that while AT successfully addresses the inability to thrive, it may not eliminate pre-existing obesity or prevent a tendency towards overeating that is already present in some children [61,62]. It also means that with a greater chance of postoperative gain, in case the patient is obese in the first place, there are risks lurking in long-term increases in weight, such as insulin resistance and high cholesterol rates, diseases deemed as even “more dangerous” by most physicians [63,64].

Sleep is an important factor in determining postoperative growth before and after AT in children [65]. OSA causes poor sleep quality, and reduced growth hormone secretion due to poor sleep quality can affect growth [66,67]. After AT, the removal of airway obstructions improves sleep quality and results in the restoration of normal GH secretion. This improvement in sleep was related to changes in height and weight z-scores, which is a marker of “catch-up growth” in children who had previously been delayed [68].

Our review uncovers that AT exacerbates pre-existing obesity, highlighting both the metabolic and surgical aspects of regulation. This process may involve several metabolic mechanisms. First, AT has a proven benefit for sleep quality by reducing airway obstruction, resulting in the enhanced secretion of both growth hormone and insulin-like growth factor-1, which are potent appetite stimulators and anabolic intermediates. Although these consequences are positive for underweight children with “catch-up growth”, they could exacerbate weight gain in already overweight or obese children [69]. Moreover, removing airway obstructions lowers the energy usage needed to overcome respiratory distress, contributing to a diminished basal metabolic rate postoperatively. Children with OSA have an elevated caloric need as a consequence of respiratory work; post-AT, the metabolism decreases but feeding habits do not, generating a caloric surplus and subsequent weight gain [70].

We need to make standardized definitions of outcome measures and find out how AT affects diverse pediatric subgroups in the long run, metabolically as well as relationally. Also, providing children with specific perioperative guidance and follow-up by nutritional counseling and weight monitoring can go a long way for optimizing surgical treatment outcomes and minimizing complications. This can provide an important reference for clinicians by integrating medical and lifestyle interventions to care for children undergoing AT and seeing to good growth, both inside and out.

### Limitations

The systematic review of our study has several limitations. The studies included had significant heterogeneity in terms of patient status, baseline weight, and operation outcomes. This prevented the combination of results into a meta-analysis that would have been more utilitarian; at the same time, it meant that various conclusions could not be drawn with assurance. One further limitation is the inability of this review to adjust for any potentially controversial factors that might mask local socio-economic indicators, like those members having undergone successful operations living more in areas where such care is rare—and perhaps also with higher levels of obesity. Of course, upon examination it was also found that factors like intracapsular versus extracapsular operation methods, the methods used by the doctors themselves, and the relative experience levels among different groups are pivotal issues that have perverse effects on the results. This naturally biased study is in favor of low-risk procedures. A lack of uniformity in operation techniques, such as differing intracapsular barrels versus extracapsular instruments, or uneven years of surgical team expertise imparted potential bias that might have influenced outcomes tabled in the available literature.

The effect that better nightly sleep can have over time is well appreciated here, but no concise research on how this relates to levels of human growth hormone (HGH) activity was ever conducted in light of adenotonsillectomy. On the whole, these limitations can be addressed through future research focusing on multi-center, large-sample studies with uniform protocols for selecting patients and mastering cutting-edge technology in surgery. Long-term follow-up studies are indispensable before this can be called an effective treatment to grow better minds or lifestyles with haste (our review found no data on any late effects). In short, statistical considerations and healthcare variables need to be weighed in balance too—or else some disparities of advantage between groups may be left undetected instead of being rectified. All these attempts will eventually offer comprehensive evidence that will serve both to refine clinical practice guidelines and to maximize outcomes for children receiving this procedure.

## 5. Conclusions

This systematic review provides extensive evidence regarding the role of AT in weight gain in children. Results suggest that AT alone was found to have a significant effect on growing outcomes, but results differed according to baseline weight status. Underweight and normal-weight children tend to show catch-up growth, with significant improvements in weight percentiles, BMI z-scores, and height growth trajectories. In contrast, overweight and obese children are more likely to gain additional weight, raising considerable concerns about the worsening of pre-existing obesity.

The variability in weight responses indicates that various physiological and metabolic mechanisms are involved, including the disruption of energy balance, sleep quality, and hormonal regulation (dysregulation of IGF-1, ghrelin, and leptin) as well as appetite regulation (higher appetite and glucose tolerance). Finally, although AT relieves upper airway obstruction and promotes growth in underweight children, the potential impact on long-term weight trajectory in obese patients requires tailored postoperative follow-up and weight management, including nutrition counseling and weight monitoring.

Furthermore, longitudinal studies and comparative trials are necessary to determine the long-term growth and metabolic effects of AT across different pediatric populations, to improve clinical practices and outcomes.

## Figures and Tables

**Table 1:** Characteristics and outcomes of studies investigating the impact of adenotonsillectomy on weight gain in children.

Authors	Country	Study Design	Patients (N)	Age	Summary	Level of Evidence
Kirkham EM [3]	Not mentioned	Observational study	458	3.0 to 12.9 years	AT was not specifically associated with an increased risk of undesirable weight gain in children with mild obstructive sleep-disordered breathing.	II
Kevat A [13]	Australia	RCT	190	Preschool-aged children, 3–5 years old	Children with mild–moderate OSA were randomly assigned to early AT surgery (within 2 months) or routine surgery (12-month wait). An increase in BMI z-scores was noticed in the early surgery group within the first 12 months after surgery but not from 12 to 24 months. Similarly, the routine surgery group showed BMI z-score increases in the 12–24 month postoperative period.	I
Esteller E [14]	Spain	Case-control study	344	Children (specific age range not mentioned)	Many children with OSA concurrent with growth failure could benefit from AT to recover and normalize their growth rate. The study found that in a one-year follow-up after surgery, 66.6% of children with height for age ≤5th percentile achieved catch-up growth.	III
Keefe KR [15]	USA	Literature review	Not mentioned	Children (specific age range not mentioned)	Obesity-associated pediatric OSA presents unique challenges, as AT is less effective in these cases. The rising prevalence of childhood obesity increases the need for personalized treatments, such as drug-induced sleep endoscopy and weight management strategies.	V
Katz ES [16]	USA	RCT	464	5 to 9.9 years	Overweight children have a higher risk of developing obesity after AT. Weight monitoring and nutritional counseling are crucial after surgery.	I
Au CT [17]	Hong Kong	RCT	114	Pre-pubertal children aged 6–11 years old	AT is associated with increased weight gain and systolic blood pressure.	I
Selimoğlu E [18]	Turkey	Prospective observational study	29	Pre-pubertal children (specific age range not mentioned)	AT improves growth in children with OSA, as evidenced by increased weight, height, energy intake, and serum IGF-1 levels 6 months after surgery.	III
Fehrm J [19]	Sweden	RCT	60	2–4 years old	Children with moderate–severe OSA are advised to undergo AT, while watchful waiting may suffice for mild cases.	I
Waters KA [20]	Australia	RCT	190	Pre-school children, 3–5 years old	AT for preschool children with OSA can improve their sleep, behavior, and OSA symptoms, regardless of the timing of surgery.	I
Ersoy B [21]	Turkey	Prospective observational study	48	3–10 years old	The study found that children with adenotonsillar hypertrophy have improved growth rate, weight, and height in the first year after surgery.	III
Waters KA [22]	Australia	RCT	210	3–5 years old	Early AT in preschool children with mild–moderate OSA can improve IQ and quality of life.	I
Chawla J [23]	Australia	RCT	190	3–5 years old	AT can improve sleep quality, behavior, and daytime functioning, as reported by parents and polysomnography.	I
Czechowicz JA [24]	USA	Retrospective review	815	18 years and younger	AT does not appear to increase obesity rates, especially if the child is already above the 80th weight percentile before surgery.	III
Kirkham EM [25]	USA	RCT	398	Children (specific age range not mentioned)	The study found that both surgical and non-surgical groups experienced similar rates of weight gain, which suggests other contributing factors.	I
Gourishetti SC [26]	USA	RCT	397	Children (specific age range not mentioned)	The study found that after AT, weight gain is mediated by improvements in REM sleep-related polysomnographic parameters, including reductions in apneic events and arousals.	I
Lee CH [27]	Taiwan	Systematic review and meta-analysis	3413	Children (specific age range not mentioned)	Non-obese children had post-surgical improvements in sleep parameters, including AHI reduction and better sleep efficiency compared to obese children.	I
Bonuck KA [28]	USA	Systematic review and meta-analysis	930	Children (specific age range not mentioned)	Standardized height, weight, and growth biomarkers (IGF-1 and IGFBP-3) in children can significantly improve after AT.	I
Kırıs M [29]	Turkey	Prospective study	96	Children (specific age range not mentioned)	AT improves weight, height, and growth biomarkers (IGF-1 and IGFBP-3) in children with sleep-disordered breathing or recurrent infections.	II
Ruiz AG [30]	USA	Retrospective chart review	78	Mean age 5.29 years	The study found that for children with Down syndrome, AT has no effect on BMI trajectory.	III

Abbreviations: AT: adenotonsillectomy; OSA: obstructive sleep apnea; RCT: randomized clinical trial; BMI: body mass index; IGF: insulin-like growth factor; IQ: intelligence quotient; REM: rapid eye movement; AHI: apnea–hypopnea index; IGFBP: insulin-like growth factor-binding protein.

**Table 2:** Comparison of postoperative growth and weight outcomes in children undergoing adenotonsillectomy.

Authors	Diagnosis and Age	Pre-Surgical Weight/BMI	Post-Surgical Weight/BMI (Follow Up 2–12 Months)	*p* Value	Interpretation
Nachalon Y [37]	OSA in children (6–36 months old)	- Weight: 11.11 ± 2.59 kg - BMI: 15.85 ± 1.67 - Z-score BMI: −0.51 ± 1.24	- Weight: 13.0 ± 2.48 kg - BMI: 16.7 ± 1.69 - Z-score BMI: 0.36 ± 1.18	0.007	Children with OSA who underwent AT have shown significantly improved weight, BMI, and z-scores, indicating “catch-up” growth after surgery.
Soultan Z [38]	OSA with enlarged tonsils in children (1.4–10.25 years)	- Mean z-score for weight: 1.37 ± 2.49 - Mean BMI score: 3.8 ± 6.4	- Mean z-score for weight: 2.0 ± 2.27 - Mean BMI score: 4.7 ± 6.7	0.004	Obese children showed further weight gain after AT, which indicates the need for weight management strategies post-surgery.
Hashemian F [39]	Chronic tonsillitis in children (2–12 years)	- BMI Percentile: 20% underweight, 67% healthy, 10% at risk, 3% overweight	- BMI Percentile: 10% underweight, 57% healthy, 22% at risk, 11% overweight	0.02	Over a period of six months, AT resulted in increases in weight, height, and BMI. Because it improved appetite but increased the risk of being overweight.
Waters KA [40]	OSA in children (6.9 ± 3.5 years)	- BMI: 19.8 ± 6.3 kg/m^2^	- BMI: 20.4 ± 5.5 kg/m^2^	Not mentioned	AT in children with OSA was associated with small improvements in cholesterol levels.
Roemmich JN [41]	OSA in children (6–12 years)	- Percentage Overweight: 32.0%	- Percentage Overweight: 36.3%	Not mentioned	AT is effective in lowering the obstructive apnea–hypopnea index from 7.6 to 0.6. However, it led to an increase in weight after surgery.
Bar A [42]	OSA in prepubertal children (6.0 ± 2.8 years)	- Weight SDS: 0.86 ± 1. - Serum IGF-I Levels: 146.3 ± 76.2 ng/mL	- Weight SDS: 1.24 ± 0.9 - Serum IGF-I Levels: 210.3 ± 112.5 ng/mL	<0.01.	AT has the potential to enhance growth in prepubertal children, as evidenced by weight SDS, which shows a significant increase 12 to 18 months after surgery.
Barr GS [43]	Recurrent tonsillitis in children (age not mentioned)	- Median weight at the 55th percentile	- Average weight increased by 20%	Not mentioned	AT in children with recurrent tonsillitis leads to a significant increase in weight and may be associated with improved appetite.
Conlon BJ [44]	Tonsillitis in children (age not mentioned)	- Mean weight was 9.8%	- Mean weight gain 12%	0.001	The study indicates that children who have weight gain after AT have improved their health and appetite.
Voora RS [45]	OSA in children (mean age: 7.6 years, SDS: 4.0)	- Median BMI percentile on the day of surgery: 65.8	- Median BMI percentile at follow-up: 76.4	0.001	The study shows an increase in BMI percentile following AT. This weight gain disproportionately affected younger, male, and preoperatively obese children.
Kang KT [46]	OSA in children (1–18 years)	- BMI >95th percentile for age and sex	- Not specifically reported	<0.001	Children with OSA have a higher risk of developing both adenoidal and tonsillar hypertrophy.
Quante M [47]	OSA in children (5 –9.9 years)	- BMI less than 2.99 z-score for age and sex	- BMI z-score increased 14%	<0.001	The study suggests that while AT may improve some cardiovascular parameters, its effects on broader cardiometabolic outcomes in children without severe hypoxemia may be limited.
Mitchell RB [48]	OSA in obese children (mean age: 9.3 years)	- Mean BMI: 28.6 (range: 19.2–47.1)	- Mean BMI: 27.9 (range: 17.8–27.9)	0.06	The study suggests that while AT effectively reduces OSA severity and improves quality of life, it may not address underlying obesity.
Levi J [49]	OSA in children (2–12 years)	- BMI percentile: *p* = 0.14	- BMI percentile: *p* < 0.001	<0.001	Young children who undergo AT typically experience weight gain. However, the weight of obese or overweight children did not significantly differ from before surgery.
Nafiu OO [50]	Tonsillitis in children (3–17 years)	- 35.1% of the patients were overweight or obese	- Not specifically reported.	<0.001	The study indicates that the heavier the child’s weight, the higher the risk of PTP.
Jeyakumar A [51]	OSA in children (age not mentioned)	- Preoperative weight ranged from normal to morbid obesity	- BMI increased by 5.5% to 8.2%	Not mentioned	AT is associated with greater-than-expected postoperative weight gain in normal and overweight children.
Nafiu OO [52]	OSA in children (age not mentioned)	- Prevalence of overweight and obesity: 20.7%	- BMI increased	<0.001	AT in overweight and obese children is associated with a higher risk of perioperative complications, including respiratory difficulties and prolonged recovery.
Chan DK [53]	OSA in children (age not mentioned)	- Preoperative weight ranged from normal to morbid obesity	- Overweight children had higher postoperative AHIs	Not mentioned	BMI and medical comorbidities are key factors affecting postoperative outcomes, suggesting careful patient selection and tailored interventions are needed.

Abbreviations: OSA: obstructive sleep apnea; AT: adenotonsillectomy; BMI: body mass index; SDS: Standard Deviation Score; IGF: insulin-like growth factor; PTP: post-tonsillectomy pain; AHIs: apnea–hypopnea indices.

## Data Availability

Not applicable due to the nature of the study.

## Abbreviations

The following abbreviations are used in this manuscript:

OSA	Obstructive sleep apnea
AT	Adenotonsillectomy
PRISMA	Preferred Reporting Items for Systematic Reviews and Meta-Analyses
MeSH	Medical subject headings
BMI	Body mass index
RCTs	Randomized controlled trials
GRADE	Grading of Recommendations Assessment, Development, and Evaluation
IGF	Insulin-like growth factor
IQ	Intelligence quotient
REM	Rapid eye movement
AHI	Apnea–hypopnea index
IGFBP	Insulin-like growth factor-binding protein
ICT	Intracapsular tonsillotomy
ECT	Extracapsular tonsillectomy
SDS	Standard Deviation Score
PTP	Post-tonsillectomy pain

## Appendix A

**Table A1:** Systematic review protocol and support template.

Title of the Systematic review	Impact of Adenotonsillectomy on Weight Gain in Children; A Systematic Review
(1) Background of the systemic review	
Adenotonsillectomy (AT) is one of the most commonly performed surgical procedures in the pediatric population. It is predominantly indicated for treating obstructive sleep apnea (OSA), recurrent tonsillitis, and chronic adenoid hypertrophy [1]. This surgical intervention has been demonstrated to considerably ameliorate airway obstruction, sleep quality, and quality of life in affected children [2]. Nevertheless, the last few years have been characterized by studies reporting an association between adenotonsillectomy and weight gain after surgery in the pediatric population, attracting wide attention and controversy [3]. The postoperative weight gain reported after AT has been related to improved feed efficiency with decreased obstruction, better quality sleep with increased growth hormone activity, and metabolic/feeding habit changes [4]. For underweight children, weight gain is a goal; however, excessive gain should be a concern in children who had a healthy weight or were overweight before surgery [5]. The association between AT and weight gain has implications for not only surgical outcomes but also long-term pediatric health outcomes, including non-infectious respiratory disease, metabolic syndrome, cardiovascular disease, and psychosocial health. Despite increased awareness, the literature on the topic is equivocal, with studies presenting differing findings depending on baseline weight status, age, gender, or presence of comorbidities [6]. Of note, variances in study designs and definitions of weight gain also make it difficult to interpret this association. The aim of this systematic review is to analyze the evidence of the effect of adenotonsillectomy on weight gain in the pediatric population by identifying trends, risk factors, and long-term consequences. This review aims to summarize the available data in an attempt to provide data-driven evidence to assist clinicians in preoperative counseling, postoperative monitoring, and general management of the pediatric population undergoing AT.	
(2) Aim of the systemic review	
This systematic review aims to assess the impact of AT on weight gain in pediatric patients, focusing on identifying the extent and patterns of postoperative weight changes. The review seeks to explore contributing factors such as baseline weight status, age, gender, and comorbidities, and to evaluate the long-term health implications of these changes. Ultimately, the review aims to provide evidence-based recommendations for preoperative counseling, postoperative care, and the management of weight-related outcomes in children undergoing adenotonsillectomy.	
(3) Specific objectives of the systemic review	
- To evaluate the extent and patterns of weight gain in children following AT. - To investigate the factors influencing postoperative weight gain, including baseline weight status, age, gender, and the presence of comorbidities. - To examine the potential long-term health implications of postoperative weight gain, including the risk of obesity, metabolic syndrome, and cardiovascular conditions. - To compare weight-related outcomes in children undergoing adenotonsillectomy for different conditions, such as OSA and recurrent tonsillitis.	
(4) Criteria for including studies in the review	
Population or participants and condition of interest	This review will include pediatric patients of different ages and genders who have undergone AT in various healthcare settings. The condition of interest in this systematic review is the impact of AT on postoperative weight gain, including its extent, contributing factors, and long-term health implications. Studies evaluating children with conditions such as OSA, recurrent tonsillitis, or chronic adenoid hypertrophy will be included.
Interventions or exposures	- Surgical Intervention: AT performed for pediatric patients with conditions such as OSA, recurrent tonsillitis, or chronic adenoid hypertrophy. The review will assess its impact on postoperative weight gain. - Baseline and Postoperative Comparisons: examination of changes in weight status, including body mass index (BMI), weight percentiles, or other growth indicators, before and after AT. - Subgroup Analysis: evaluation of the influence of various factors on weight outcomes, including age, gender, baseline weight status (underweight, healthy weight, overweight), and the presence of comorbidities such as OSA or metabolic disorders. - Lifestyle and Dietary Factors: assessment of potential lifestyle or dietary habit changes post-surgery and their contribution to weight gain.
Comparisons or control groups	Standard Care: pediatric patients with similar conditions (e.g., OSA, recurrent tonsillitis, or adenoid hypertrophy) who did not undergo AT.
Outcomes of interest	- Primary Outcomes: Postoperative weight gain (measured as absolute weight change, BMI change, or growth percentile shift). Incidence of excessive weight gain or obesity after AT. - Secondary Outcomes: Long-term health implications of postoperative weight gain, including risks of obesity, metabolic syndrome, and cardiovascular conditions. Differences in weight-related outcomes based on baseline weight status, age, gender, and comorbidities. Impact of weight gain on overall pediatric growth and development. - Patient-Centered Outcomes: Quality of life changes associated with postoperative weight gain (e.g., psychosocial impacts, self-esteem). Parental or caregiver concerns regarding weight changes after surgery. - Healthcare-Related Outcomes: Postoperative interventions or counseling required to manage weight gain. Burden on healthcare systems associated with managing long-term effects of postoperative obesity.
Setting	- Hospitals: general hospitals and specialized pediatric centers where adenotonsillectomy is performed and postoperative care is provided. - Outpatient clinics: healthcare facilities where children are evaluated preoperatively for conditions like obstructive sleep apnea or recurrent tonsillitis and followed up postoperatively for growth and weight monitoring. - Community healthcare settings: primary care or community health centers where ongoing management, counseling, and nutritional guidance are provided to children post-adenotonsillectomy. - Long-term care facilities: rehabilitation centers or other extended care facilities for children with complications from excessive weight gain or other comorbid conditions post-surgery.
Inclusion criteria	Studies that investigate the impact of adenotonsillectomy on postoperative weight gain in pediatric patients. Studies involving participants aged 0–18 years who have undergone adenotonsillectomy for conditions such as obstructive sleep apnea, recurrent tonsillitis, or adenoid hypertrophy. Studies reporting quantitative data on outcomes of interest, such as weight gain, BMI changes, growth percentiles, or associated health implications. Case-control studies, cohort studies, randomized controlled trials (RCTs), and before–after studies are included. Studies published in the English language.
Exclusion criteria	Studies that do not focus on pediatric patients (ages 0–18 years) undergoing adenotonsillectomy. Studies that do not report outcomes related to weight gain, BMI changes, or growth patterns post-surgery. Animal studies, in vitro studies, and review articles. Studies published in languages other than English. Studies with insufficient data or lack of relevant outcome measures.
(5) Search methods	
Electronic databases	- PubMed database - Web of science - Embase - Cochrane Library
Keywords	(Adenotonsillectomy OR Weight gain OR Pediatric surgery OR Obstructive sleep apnea OR Recurrent tonsillitis OR Growth outcomes OR Postoperative complications OR Pediatric otolaryngology)
(6) Methods of review	
Details of methods, number of reviewers, how agreements are to be reached and disagreements dealt with, etc.	Three primary reviewers and a fourth to adjudicate any disputes. Dr. Mohammed and Dr. Arwa address any unresolved disputes in an article.
Quality assessment tools or checklists used with references or URLs	The protocol will delineate the approach for literature critique/appraisal and will employ the STROBE tool to assess the pertinent content and technique of each paper under evaluation.
Narrative synthesis details of what and how synthesis will be done	Narrative synthesis will occur concurrently with any meta-analysis, utilizing a framework comprising four elements: 1—Formulating a hypothesis regarding the mechanism of the intervention, its rationale, and its target population. 2—Formulating an initial synthesis of the findings from the listed studies. 3—Investigating links both within and among studies. 4—Evaluating the resilience of the synthesis.
Meta-analysis details of what and how analysis and testing will be done. If no meta-analysis is to be conducted, please give reason.	A meta-analysis is anticipated; however, its realization will depend on the data gathered and made accessible from the systematic review. It is necessary to consider the exploration of heterogeneity.
Grading evidence system used, if any, such as GRADE	GRADE will be employed for the evaluation of evidence.
(7) Presentation of results	
Additional material summary tables, flowcharts, etc., to be included in the final paper	Comprehensive flowchart of the entire procedure Procedure Extraction of data from tables Forest plots of studies incorporated in the final review

## Appendix B

**Table A2:** The Newcastle–Ottawa Scale for the included cohort prospective and retrospective studies.

	Selection				Comparability	Outcome			Quality Score	Risk of Bias (0-3: High, 4-6: Moderate, 7-9: Low)
Article	Q1	Q2	Q3	Q4	Q5	Q6	Q7	Q8		
Jaensch, 2024 [1]	(A) truly representative	(A) derived from the same population	*	*	One factor controlled (age)	*	Yes	*	Moderate Quality Study (6)	Moderate Risk
Kirkham, 2024 [3]	(A) truly representative	(A) derived from the same population	**	**	Two factors controlled (age and weight)	**	Yes	**	Good Quality Study (8)	Low Risk
Ruiz, 2019 [30]	(A) truly representative	(A) derived from the same population	**	**	Two factors controlled (Down Syndrome and obesity risk)	**	Yes	**	Good Quality Study (7)	Low Risk
Nathan, 2021 [32]	(A) truly representative	(A) derived from the same population	**	**	One factor controlled (BMI z-score)	*	Yes	*	Good Quality Study (7)	Low Risk
Mesolella, 2023 [33]	(B) somewhat representative	(B) drawn from the same community	**	**	Two factors controlled (OSA and tonsil type)	**	Yes	**	Good Quality Study (8)	Low Risk
Williams, 1991 [34]	(B) somewhat representative	(B) drawn from the same community	**	**	Two factors controlled (age and weight)	**	Yes	**	Good Quality Study (8)	Low Risk
Fernandes, 2008 [35]	(A) truly representative	(A) derived from the same population	**	**	Two factors controlled (growth and weight)	**	Yes	*	Good Quality Study (7)	Low Risk
Barr, 1988 [43]	(C) selected group	(C) no description of the non-exposed cohort	*	*	No comparability	*	Yes	*	Poor Quality Study (4)	Moderate Risk
Lee, 2020 [55]	(B) somewhat representative	(A) derived from the same population	**	**	One factor controlled (age)	*	Yes	*	Moderate Quality Study (6)	Moderate Risk

Selection: Q1. Representativeness of the exposure cohort? Q2. Selection of the non-exposure cohort? Q3. Ascertainment of exposure? Q4. Demonstration that outcome of interest was not present at the start of the study?; Comparability: Q5. Comparability of cohort based on the design or analysis?; Outcome: Q6. Assessment of outcome? Q7. Was follow-up long enough for outcomes to occur? Q8. Adequacy of follow-up of cohorts? *Means the corresponding quality component or criterion is partially met, ** means the corresponding quality component or criterion is fully met.

## Appendix C

**Table A3:** Bias of the included cross-sectional studies evaluated according to the Newcastle–Ottawa Scale.

	Selection				Comparability	Outcome		Quality Score	Risk of Bias (0–3: High, 4–6: Moderate, 7–9: Low)
	Q1	Q2	Q3	Q4	Q5	Q6	Q7		
Bonuck, 2008 [28]	*	*	*	*	*	*	*	Good Quality Study (7)	Low Risk
Roemmich, 2006 [41]	*	*	*	*	*	*	*	Moderate Quality Study (6)	Moderate Risk
Bar, 1999 [42]	**	*	**	**	*	**	**	Good Quality Study (8)	Low Risk
Kang, 2013 [46]	**	*	**	**	**	**	**	Good Quality Study (8)	Low Risk
Quante, 2015 [47]	*	*	*	**	*	*	*	Moderate Quality Study (6)	Moderate Risk
Mitchell, 2004 [48]	**	**	**	**	**	**	**	Good Quality Study (9)	Low Risk
Levi, 2012 [49]	*	*	**	*	*	**	**	Good Quality Study (8)	Low Risk
Kang, 2020 [45]	**	**	**	*	**	**	**	Good Quality Study (8)	Low Risk

Selection: Q1. Representativeness of the exposure cohort? Q2. Sample size? Q3. Ascertainment of exposure? Q4. Response rate? Q5. Ascertainment of the screening/surveillance tool?; Comparability: Q5. The potential confounders were investigated by subgroup analysis or multivariable analysis?; Outcome: Q6. Assessment of outcome?; Q7. Statistical test?; * Means the corresponding quality component or criterion is partially met, ** means the corresponding quality component or criterion is fully met.

## Appendix D

**Table A4:** The Cochrane Risk of Bias assessment tool for randomized trials (RoB 2).

Articles	Bias Arising from the Randomization Process	Bias Due to Deviations from Intended Interventions	Blinding of Participants and Personnel	Bias Due to Missing Outcome Data	Bias in Measurement of the Outcome	Bias in Selection of the Reported Result	Overall RoB
Blakely, 2024 [5]	Low Risk	Low Risk	Low Risk	Low Risk	Low Risk	Low Risk	Low Risk
Choo, 2022 [6]	Low Risk	Low Risk	Low Risk	Low Risk	Low Risk	Low Risk	Low Risk
Katz, 2014 [16]	Low Risk	Low Risk	Low Risk	Low Risk	Low Risk	Low Risk	Low Risk
Au, 2021 [17]	Low Risk	Low Risk	Low Risk	Low Risk	Low Risk	Low Risk	Low Risk
Fehrm, 2020 [19]	Low Risk	Low Risk	Low Risk	Low Risk	Low Risk	Low Risk	Low Risk
Waters, 2021 [20]	Low Risk	Low Risk	Low Risk	Low Risk	Low Risk	Low Risk	Low Risk
Waters, 2020 [23]	Low Risk	Low Risk	Low Risk	Low Risk	Low Risk	Low Risk	Low Risk
Czechowicz, 2014 [24]	Low Risk	Low Risk	Low Risk	Low Risk	Low Risk	Low Risk	Low Risk
Han, 2023 [36]	Low Risk	Low Risk	Low Risk	Low Risk	Low Risk	Low Risk	Low Risk
Kang, 2008 [62]	Low Risk	Low Risk	Low Risk	Low Risk	Low Risk	Low Risk	Low Risk
Aydogan, 2007 [57]	Low Risk	Low Risk	Low Risk	Low Risk	Low Risk	Low Risk	Low Risk
